# Frequencies of an *IFNL4* Variant in an Admixed Population from Amazonia and Its Influence on Hepatitis C Infection

**DOI:** 10.3390/ijms252312764

**Published:** 2024-11-27

**Authors:** Carolina Cabral Angelim, Álesson Adam Fonseca Andrade, Renata Santos de Sousa, Raissa Lima Correa, Amanda Roberta Vieira Sacramento, Letícia Dias Martins, Simone Regina Souza da Silva Conde, Antonio Carlos Rosário Vallinoto, Rosimar Neris Martins Feitosa, Greice de Lemos Cardoso Costa

**Affiliations:** 1 Laboratory of Human and Medical Genetics, Federal University of Pará, Belém 66073-000, Brazil; carolina.angelim@icb.ufpa.br (C.C.A.); alesson.andrade@icb.ufpa.br (Á.A.F.A.); raissa.correa@icb.ufpa.br (R.L.C.); lemartias@hotmail.com (L.D.M.); 2 Virology Laboratory, Federal University of Pará, Belém 66073-000, Brazil; renata.sousa@ics.ufpa.br (R.S.d.S.); vallinoto@ufpa.br (A.C.R.V.); rosimar@ufpa.br (R.N.M.F.); 3 Postgraduate Program in Virology, Evandro Chagas Institute, Ananindeua 67030-000, Brazil; roberta.amanda@hotmail.com; 4 Faculty of Medicine, Institute of Health Sciences, Federal University of Pará, Belém 66055-080, Brazil; sconde@ufpa.br

**Keywords:** hepatitis C, *IFNL4*, SNPs, fibrosis, correlation

## Abstract

The rs12979860 polymorphism, related to the *IFNL4* gene, is suggested as a factor that impacts fibrosis progression in hepatitis C virus (HCV) infection and exhibits a wide distribution pattern across global populations. In this retrospective cross-sectional study, we aimed to investigate the frequency of this variant in an Amazonian population from Brazil, as well as its association with liver fibrosis development and its staging in HCV carriers. Our results show a significant association of the TT genotype in the sample of patients with HCV (OR = 2.291; 95% CI = 1.088–4.826; *p* = 0.033) and the greater frequency of the T allele (62.1%), which is similar to the those of African populational groups. Populational genetics analysis showed significant differences in allele frequencies on global levels. The frequency of the C allele in the study population (37.8%) was like that of the African population (39.7%), and differed from all other populations, which ranged from 62.5% to 92.9%. These findings suggest that rs12979860 plays a role in susceptibility to hepatitis C. Additionally, they allow us to propose that the response to hepatitis C infection in this group may resemble that of the African population.

## 1. Introduction

The *IFNL4* gene, which encodes the interferon lambda 4 protein, plays a crucial role in the body’s immune response, particularly in the context of viral infections such as the Hepatitis C Virus (HCV) [1]. Research has identified several genetic variants within the *IFNL4* gene that significantly influence the effectiveness of HCV clearance and response to antiviral treatments [2]. Among the most studied, rs12979860 is among the polymorphisms that have demonstrated strong associations with both HCV clearance and the progression of liver fibrosis [3]. Understanding the genetic landscape of this variant provides insight into its clinical significance.

The rs12979860 variant, located on the first intron of the *IFNL4* gene on chromosome 19, was one of the earliest discovered polymorphisms associated with spontaneous HCV clearance and treatment outcomes [4]. Individuals carrying the C allele, particularly those with the CC genotype, are more likely to clear the virus naturally and respond better to interferon-based therapies. Conversely, individuals with the T allele (CT or TT genotypes) show a reduced likelihood of viral clearance and are at higher risk of developing chronic HCV infection [5]. This variant brings forth the importance of discussing genetic predisposition and has been widely studied across diverse populations.

The relationship between this *IFNL4* variant and fibrosis development is of clinical interest. Liver fibrosis, a common consequence of hepatitis C, can progress to cirrhosis or hepatocellular carcinoma (HCC) if left untreated [6]. Studies have demonstrated that individuals with unfavorable genotypes for rs12979860 not only struggle with viral clearance but are also at an increased risk of developing severe liver fibrosis [7]. These findings underscore the dual role of *IFNL4* gene variants in shaping both antiviral responses and fibrotic processes within the liver, making them important genetic markers for predicting long-term liver health in HCV-infected patients.

The genotypic frequencies of rs12979860 vary significantly across different global populations, reflecting evolutionary pressures and the distinct epidemiology of HCV. For instance, the favorable rs12979860 CC genotype is more common in European and East Asian populations, while the less favorable TT genotype is more prevalent among individuals of African descent [8,9]. Given the extensive process of miscegenation within the Brazilian population, it is challenging to determine which groups with hepatitis C may exhibit a more favorable or unfavorable prognosis. Therefore, this study aimed to evaluate the genotypic frequency of the rs12979860 variant in an Amazonian population in Brazil and their correlation with fibrosis in patients with hepatitis C.

## 2. Results

### 2.1. Populational Description

The study population includes 106 participants chronically infected with HCV. These individuals were compared to a control group of 85 people described in the study of Amaral [10]. Gender-wise, there was a prevalence of men compared to women (57/49). When discussing age, 90.6% (96/106) of all patients were over 40 years old. Other sociodemographic information is presented in Supplementary Table S1.

Biochemical tests showed that over 80% of individuals presented results within the reference values range for albumin, and more than 60% were above the reference values for AST, ALT, GGT and FA. Regarding viral load, approximately 68% of carriers had a viral load below 8 *×* 10^5^ IU/mL while 32% were above this value, which is considered a high viral load [11]. More than 70% of the total number of infections were caused by genotype 1, followed by genotype 3 (27%) and the remainder by the other genotypes described in the literature.

**Figure 1:**
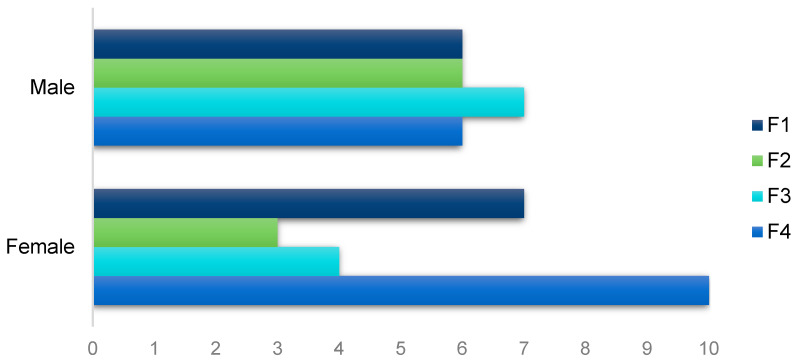
Stage of fibrosis according to the sex of HCV-infected patients.

Approximately 46.2% (49/106) of patients developed fibrosis (score F1–F4 on the METAVIR scale) and 32.6% (16/49) of them developed cirrhosis (F4). Although there is no statistical difference (*p*-value = 0.411), we observed that mild (F0–F2) and moderate (F3) fibrosis were more frequent in men and that advanced fibrosis (F4) was more frequent in women (Figure 1). We performed a comparison between data from mild fibrosis (F0–F2) and advanced fibrosis (F3–F4) groups, but none of them was statistically significant (Supplementary Table S2).

Table 1 shows the baseline clinical characteristics of patients with hepatitis C. It is observed that there are no differences between the groups with and without fibrosis in terms of sociodemographic (sex and age) characteristics. The group infected by HCV genotype 1 presented a greater susceptibility to developing hepatic fibrosis (OR = 3.75, 95% CI = 1.03–13.6, *p* = 0.039). On biochemical tests, only the AST enzyme was higher in the group with fibrosis (*p*-value = 0.012). 

### 2.2. Genetic Characterization

Both groups –HCV and Control– presented a higher prevalence of heterozygous individuals (CT). In the HCV group, 70% (74/106) of them were categorized as heterozygous, 27.2% (29/106) were homozygous for the T allele (TT) and 2.8% (3/106) were homozygous for the C allele (CC). On the other hand, in the control group, the CC genotype was quantitatively the second highest, while the TT genotype was the least frequent (Table 2). These frequencies were then compared and indicated that the CC and CT genotypes are more frequent in samples from healthy individuals (control group) and HCV carriers, respectively, which suggests a greater possibility of association between the TT variant genotype and the HCV viral infection (OR = 2.291; 95% CI = 1.088–4.826; *p* = 0.033).

Regarding the allelic frequency, the T allele was present in 62% of the HCV group. The control group, as it turns out, presented the same frequency percentage, but for the C allele. This notable difference highlights the protective role of the reference allele against the virus in these individuals. Hardy–Weinberg disequilibrium (HWE) was also observed in the HCV group, and this is possibly due to the low frequency of the CC genotype (*p*-value ≤ 0.001).

### 2.3. Genetic Correlation with Fibrosis

The genotypic frequencies of rs12979860 were compared in the group without fibrosis vs. with fibrosis and mild fibrosis (F0–F2) vs. advanced fibrosis (F3–F4). It was observed that there is no correlation between the genotypes of this variant and fibrosis development or its staging (Table 3). There are also no differences between the groups regarding allelic frequencies since they all presented an increased prevalence of the T allele. We also made a comparison between those groups, adopting the recessive model (TT versus CC + CT). However, none of the results was significant (Supplementary Table S3).

### 2.4. Populational Genetics

**Figure 2:**
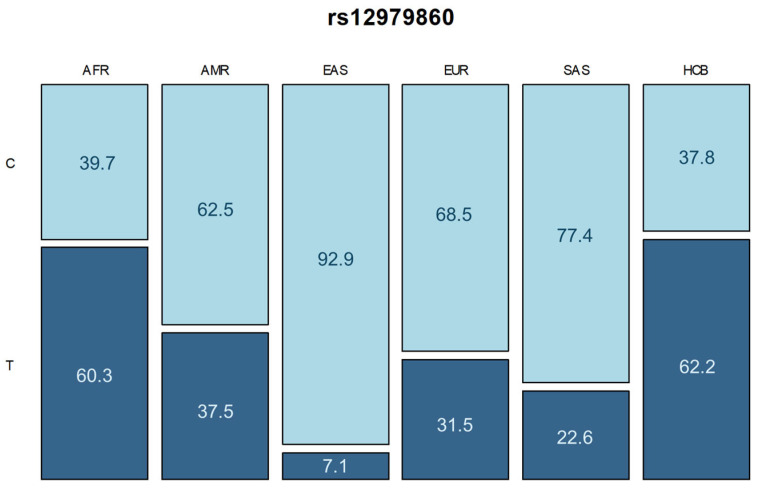
Population comparison between allele frequencies of the rs12979860 variant. Dark blue represents the T allele frequencies and light blue represents the C allele frequencies.

The distribution of this *IFNL4* variant in the sample of HCV carriers was shown in Figure 2, along with the allelic frequencies in each of the five continental population groups described by GnomAD v3.1.2—Africa, Americas, East Asia, Europe, and South Asia in percentage (%). Comparison between the sample of HCV carriers from Belém (HCB) and data from GnomAD shows that our sample differs from AMR (*p*-value: 0.0018), EAS, EUR and SAS (*p*-value: <0.0001). Below, Table 4 presents all *p*-values obtained during comparative analyses between populations.

## 3. Discussion

The association of *IFNL4* gene variants with different clinical outcomes has been investigated for at least 10 years in GWAS studies [12]. Several authors have already demonstrated that SNVs of this gene are related to the spontaneous elimination of HCV [13,14], response to antiviral therapies [5] and development of liver complications [8,15,16]. In our study, we approached the frequencies of the rs12979860 variant and evaluated their interference in the development of fibrosis.

There was a higher prevalence of men in relation to women, like the findings of other studies that mention greater exposure of this group [17,18]. In addition, age did not indicate a greater propensity for advanced fibrosis, although other studies have shown the opposite [15,19]. In biochemical tests, we observed an increase in liver enzymes in most individuals, especially AST in the group with fibrosis. Both results are consistent with the infectious and inflammatory panorama previously demonstrated in other studies [20,21].

The genotypic analysis showed a higher frequency of the TT genotype in the sample of HCV carriers when compared to the population without this infection. Similar results are mentioned by Mohammad [22] and Abd Alla [23], which emphasize the association of rs12979860TT genotype with HCV susceptibility and treatment outcome, greater hepatic changes (cirrhosis), and an increased likelihood of serological relapse. The Hardy–Weinberg disequilibrium observed in our study is also mentioned in the studies of Silva [24] and Magri et al. [25], both justifying it by the reduced frequency of the rs12979860-CC genotype as well as presented in this study.

Regarding the allele frequencies, the population of this study presented a higher prevalence of T allele (62%). Similar results were found by Conde [26] and Cavalcante [27] in northern and northeastern populations from Brazil, in which T allele frequency was 51.3% and 54.7%, respectively. However, our result differs from those of Nastri [19] and Costa e Silva [28] in southeastern and Central Brazil populations, which reported the prevalence of the C allele (63% and 52.5%, respectively).

It is important to consider that Brazil is a country of continental dimensions, and the differences found are certainly a reflection of our populational heterogeneity, due to the colonization process. This has resulted in a high level of ethnic diversity, as well as a widely variable genetic profile in different regions [29,30]. This aspect is also highlighted in the populational genetics analysis, which identified a similarity between HCV carriers from Belém (HCB) and African populations. Nevertheless, our study population differed from American, European, and Asian populations.

The contemporary Brazilian population, especially those from the Amazon region, have strong European, Amerindian, and African ancestry [31,32]. Studies demonstrate that the T variant allele is the most frequent in African populations [8,9]. Thus, the similarity between the allelic frequencies of the group of this study and that of African populations is coherent due to its role in forming the Amazonian Brazilian population [29,31]. The similarities between *IFNL4* frequencies also suggest that the sample of HCV carriers collected in this study may have randomly selected, through sociodemographic characteristics, a profile of individuals with prevalent genetic characteristics of African origin.

The absence of association between rs12979860 and the development of fibrosis and its severity in the Amazonian population of Brazil with hepatitis C is similar to other studies in mixed-race populations in the state of Pará [10,33]. However, they differ from studies by Eslam [34] and Bibert [35], carried out with more ethnically homogeneous populations (Caucasians and Swiss, respectively). This was expected since the samples of these two studies are predominantly European groups, where the allelic frequency differs from our sample.

We believe that the sample size represents the greatest limitation of this study and therefore is an aspect that can be further explored in subsequent studies with the same approach. Despite this, our results (i) emphasize the importance of the rs12979860 variant in the context of susceptibility to HCV, (ii) suggest a significant role of world populations in the formation of the genetic profile of the Amazonian Brazilian population and (iii) point out the lack of association between this *IFNL4* variant and fibrosis presence and its severity. Finally, it is understood that this study can aid the creation of public policies and strategies for combatting hepatitis C in this population.

## 4. Materials and Methods

### 4.1. Study Population

This observational, cross-sectional, and retrospective study investigated 106 individuals chronically infected with HCV, with samples acquired through partnership with three healthcare services: Hospital Universitário João de Barros Barreto (HUJBB), Centro de Atenção à Saúde em Doenças Infecciosas Adquiridas (CASADIA), and Fundação Santa Casa de Misericórdia do Pará (FSCMPA). The samples used in this study from FSCMPA were collected between March 2014 and December 2016; samples from HUJBB and CASADIA were collected between 26 May 2023 and 8 December 2023.

This study was approved by the National Human Research Ethics Committee and the Research Ethics Committee from the Institute of Biological Sciences of the Federal University of Pará (CAE: 65072922.6.0000.0018). This study also received ethical approval from the ethics committees within the institutions involved. The participation of all individuals was conditional on the acceptance and signing of an informed consent form (ICF) and the sociodemographic questionnaire prepared for the research.

The inclusion criteria used to consider individuals eligible for this research were (i) to be over 18 years, (ii) reactive serology for total anti-HCV antibodies, (iii) viral RNA detectable by RT-PCR, (iv) a signed informed consent form and (v) completion of the sociodemographic questionnaire. The exclusion criteria were (i) coinfection with other viruses with hepatic tropism, (ii) lack of laboratory information (which would limit statistical analysis), (iii) not adequately answering the sociodemographic questionnaire and (iv) they did not initiate any treatment until the moment of collection.

The control group consists of 85 individuals from the Metropolitan region of Belém, State of Pará, Brazil, assisted on Santa Casa de Misericórdia do Pará. The group was composed of individuals of both sexes, over 18 years, with a non-reactive serology for total anti-HCV antibodies, and clinical and laboratory characteristics within reference values by medical professionals.

### 4.2. Laboratory Tests

To assess liver injury (necroinflammatory activity), the biochemical markers aspartate aminotransferase (AST) and alanine aminotransferase (ALT) were used. To evaluate bile flow, gamma-glutamyl transpeptidase (GGT) and alkaline phosphatase (FA) were adopted, and liver function was evaluated by albumin (ALB) values. HCV-RNA quantification was carried out using the COBAS 5800 HCV test diagnostic kit (Roche, Indianapolis, IN, USA) and HCV genotype research was carried out using reverse hybridization using nitrocellulose strips from the INNO-LiPA HCV kit. II (Immunogenetic, Carlsbad, CA, USA).

### 4.3. Histopathologic Exam

At first, the individuals underwent abdominal ultrasound examination to evaluate changes in liver morphology. Based on medical evaluation, patients with indicative signs of fibrosis underwent liver biopsy to verify periportal activity and the fibrosis stage according to the METAVIR classification. This procedure followed the FSCMPA liver outpatient protocol, and the staging followed the standardization established by Bedossa and Poynard [36] as shown in Table 5. All data were tabulated in Excel. Data from FSCMPA were accessed on 19 February 2023 and the database with HUJBB and CASADIA medical information on 19 January 2024

### 4.4. Molecular Analysis

In the DNA extraction and purification stage, 200 μL of whole blood was used, which was subjected to DNA extraction using the PureLink^®^ Genomic DNA mini kit (Invitrogen, Thermo Fisher Scientific, Waltham, MA, USA). The protocol defined by the manufacturer was used, which includes 2 steps: DNA isolation and purification. The integrity of the samples was assessed by electrophoresis in a 1.5% agarose gel and quantification was performed on the NanoDrop™ 2000/2000c spectrophotometer (Thermo Fisher Scientific, MA, USA), resulting in samples containing at least 20 ng of DNA.

In the genotyping stage, the identification of polymorphisms occurred through amplification of the genetic material by real-time PCR (qPCR), according to the manufacturer’s protocol on the Applied Biosystems 7500 equipment (Thermo Fisher, Carlsbad, CA, USA). TaqMan™ Universal PCR Master Mix reagents and fluorescent probes (Thermo Fisher, Carlsbad, CA, USA) were used for targets rs12979860 (ID: C___7820464_10: a predesigned TaqMan SNP Genotyping Assay) of the *IFNL4* gene, whose sequences are found in Table 6. The protocol used consists of an adaptation of the manufacturer’s protocol, in which each well of the microplate (capacity 100 μL) contained 8 μL.

### 4.5. Statistical Analysis

Genotypic and allele frequencies were obtained by simple counting, and comparisons between groups were performed using Fisher’s exact test, with the odds ratio and a 95% confidence interval (CI) as measures of association. The sample from this study was compared to a control group described in a study published by Amaral [10]. This group was chosen due to its similarities with our sample: (i) population substructuring, (ii) same location—Belém, State of Pará, Brazil and (iii) same institution of health assistance—FSCMPA. These characteristics reduce the risk of false significance in our analyses.

For the statistical analysis, four groups were established: fibrosis, no fibrosis, mild fibrosis (F0–F2), and advanced fibrosis (F3–F4). The normality of the laboratory data (AST, ALT, GGT, FA, albumin, and viral load) was verified using the Shapiro–Wilk test, and the differences between the groups were analyzed using the Mann–Whitney U test. Differences between groups for categorical data (sex, age, and HCV genotype) were assessed using the Chi-square (χ^2^) test, as well as the Hardy–Weinberg equilibrium test.

Differences in allele frequencies between the population in this study and those presented by GnomAD populations (AFR, AMR, EAS, EUR, and SAS) were compared using Fisher’s exact test. The Benjamini–Hochberg false discovery rate (FDR) test was applied as a statistical correction for multiple comparisons. Clinical information and genotyping data were stored in a database using Microsoft Excel 365 software. All tests were conducted using R Studio v.4.3.2 and Jamovi v.2.3 programs, with a statistical significance threshold set at 95% (*p* < 0.05).

## Figures and Tables

**Table 1:** Clinical characteristics of HCV carriers according to the presence of fibrosis.

Variables	Total *n* = 106	No Fibrosis *n* = 57	Fibrosis *n* = 49	*p*
Sex (male/female)	57/49	32/25	25/24	0.598 ^a^
Age (years)	56 (25–85)	56 (25–80)	56 (31–85)	0.279 ^a^
HCV genotype (1/3)	37/14	12/9	25/5	**0.039** ^a^
Viral Load (IU/mL)	4.1 × 10^5^ (1.7 × 10^5^–4.2 × 10^7^)	4.8 × 10^5^ (1.7 × 10^1^–4.2 × 10^7^)	3.7 × 10^5^ (5.6 × 10^3^–4.2 × 10^6^)	0.881 ^b^
AST (U/L)	55.5 (17–351)	45 (17–182)	65 (19–351)	**0.012** ^b^
ALT (U/L)	58.5 (13–322)	51 (20–212)	70 (13–322)	0.051 ^b^
GGT (U/L)	62 (12–984)	69 (17–984)	59.5 (12–244)	0.794 ^b^
FA (U/L)	146 (2.72–661)	183.9 (2.72–661)	113.5 (35–336)	1 ^b^
ALB (g/dL)	4.3 (2.9–6.5)	4.4 (3.1–5.1)	4.2 (2.9–6.5)	0.949 ^b^

Information regarding the HCV genotype was available only for 51 samples, out of the total 106. Bold formatting represents a significant difference between groups. Categorical variables are expressed as absolute values and numerical variables are expressed as median (minimum-maximum). AST = aspartate aminotransferase, ALT = alanine aminotransferase, GGT = gamma-glutamyl transpeptidase, FA = alkaline phosphatase, and ALB = albumin. ^a^ Chi-square test (χ^2^); ^b^ Mann–Whitney U test.

**Table 2:** Genotypic and allelic frequencies of the rs12979860 variant in HCV and control groups.

SNP	Genotypes	HCV Group *n =* 106 (%)	Control Group *n =* 85 (%)	OR (95% CI)	*p*
rs12979860	CC	3 (3)	32 (38)	2.291 (1.09–4.83)	**0.033**
	CT	74 (70)	41 (48)		
	TT	29 (27)	12 (14)		
Alleles	C *	80 (38)	105 (62)		**0.001**
	T	132 (62)	65 (38)		
	Total	212 (100)	170 (100)		
	HWE	<0.0001	0.981		

* Represent the reference allele. Bold formatting represents significant differences on Fisher’s exact test (*p*-value < 0.05). HWE = Hardy–Weinberg Equilibrium.

**Table 3:** Correlation between rs12979860 genotypes and fibrosis presence and its staging.

rs12979860	No Fibrosis *n* = 57 (%)	Fibrosis *n* = 49 (%)	*p*	Fibrosis F0–F2 *n* = 22 (%)	Fibrosis F3–F4 *n* = 27 (%)	*p*
CC	2 (3)	1 (2)		0 (0)	1 (4)	
CT	41 (72)	33 (67)	0.802	17 (77)	19 (70)	1
TT	14 (25)	15 (31)		5 (23)	7 (26)	
C *	45 (39)	35 (44)	0.551	17 (39)	21 (39)	0.980
T	69 (61)	45 (56)		27 (61)	33 (61)	

* Represent the reference allele. *p*-value refers to Fisher’s exact test.

**Table 4:** Paired comparison (*p*-value) of allele frequencies observed in the population of HCV carriers from Belém to the Gnomad populations, for the rs12979860 variant.

	*p*	Adjusted *p*
AFR vs. HCB	0.8848	0.8848
AMR vs. HCB	0.0011	0.0018
EAS vs. HCB	<0.0001	<0.0001
EUR vs. HCB	<0.0001	<0.0001
SAS vs. HCB	<0.0001	<0.0001

Shaded cells represent significant differences between the two populations.

**Table 5:** Histopathological classification METAVIR score.

Score	Histological Activity	Stage	Fibrosis
A0	Absent	F0	No fibrosis
A1	Mild	F1	Portal fibrosis without septa
A2	Moderate	F2	Portal fibrosis with few septa
A3	Severe	F3	Numerous septa. No nodules
		F4	Complete nodules: cirrhosis

**Table 6:** Sequences of the probes used in the qPCR reaction.

Probe ID	Fluorescence	Sequence 5′-3′
rs12979860	VIC	TGAACCAGGGAGCTCCCCGAAGGCGCGAACCAGGGTTGAATTGCACTCCGC
	FAM	TGAACCAGGGAGCTCCCCGAAGGCGTGAACCAGGGTTGAATTGCACTCCGC

## Data Availability

The original contributions presented in this study are included in this article, and further inquiries can be directed to the corresponding author.

## Supplementary Materials

The supporting information can be downloaded at: https://www.mdpi.com/article/10.3390/ijms252312764/s1.
