# Supplementary material for: Frequencies of an IFNL4 Variant in an Admixed Population from Amazonia and Its Influence on Hepatitis C Infection
Source: Int J Mol Sci. 2024 Nov 27;25(23):12764. doi: 10.3390/ijms252312764 (PMC11640799; doi:10.3390/ijms252312764)
Supplement: Supplementary file 1 [file ijms-25-12764-s001.zip › ijms-3310807-supplementary.pdf]

SUPPLEMENTARY MATERIAL

Article: Frequencies of an *IFNL4* variant in an admixed population from Amazonia and its influence on hepatitis C infection.

Supplementary Table S1. Sociodemographic characterization of people with HCV.

	HCV group *n*=106 (%)	
	Yes	No
**Risk factors in liver diseases**		
Smoking	10 (0.1)	96 (99.9)
Alcoholism	28 (26.4)	78 (73.6)
**Risk factors for HCV acquisition**		
Reuse of medical equipment*	33 (31.1)	73 (68.9)
Injectable drugs	26 (24.5)	80 (73.6)
Inhalant drugs	28 (26.4)	78 (73.6)
Blood transfusion		
Before 1993	26 (24.5)	80 (73.6)
After 1993	14 (13.2)	92 (86.8)
Tattoo	21 (19.8)	85 (80.2)
**Comorbidities**		
Arterial hypertension	18 (17)	88 (83)
Diabetes mellitus	9 (8.5)	97 (91.5)
Dyslipidemia	4 (3.8)	102 (96.2)

*Glass syringes in laboratory and hospital environments.

Supplementary Table S2. Comparison between groups with mild (F0-F2) and advanced (F3-F4) fibrosis (*p-value*) regarding clinical data.

Variables	Fibrosis F0-F2 *n* (%)	Fibrosis F3-F4 *n* (%)	*P*
**Sex**			
Male	12 (54.5)	13 (48.1)	0.656[a]
Female	10(45.5)	14 (51.9)	
**Age**			
<40 years	2 (0.1)	1 (3.7)	0.474[a]
≥40 years	20 (90.9)	26 (96.3)	
**Viral genotype**			
1	17 (81)	15 (68.2)	0.337[a]
3	4 (19)	7 (31.8)	
**Viral Load**			
≤8E+05	14 (73.7)	19 (86.4)	0.315[a]
>8E+05	5 (26.3)	3 (13.6)	
**Laboratory tests**			
AST (U/L)	61 (38.5-102.5)	58 (37-77.5)	0.657[b]
ALT (U/L)	58 (35.5-117)	62.5 (31-115)	0.562[b]

GGT (U/L)	67 (39.5-142)	50 (38-84)	0.859[b]
FA (U/L)	127 (94-189)	124 (100-149)	0.654[b]
ALB (g/dL)	4.4 (4.1-4.6)	4.1 (3.6-4.3)	0.992[b]

Categorical variables expressed as absolute (percentage) values and numerical variables expressed as median (25th quartile-75th quartile). [a] Chi-square (X^2) test, [b] Mann-Whitney test

Supplementary Table S3. Correlation between rs12979860 genotypes and fibrosis presence and its staging.

**rs12979860**	**No Fibrosis** *n* = 57 (%)	**Fibrosis** *n* = 49 (%)	**OR (95% CI)**	***P***
TT	14 (24.5)	15 (30.6)	1.355 (0.576-3.190)	0.519
CC+CT	43 (75.5)	34 (69.4)		
	**Fibrosis F0-F2** *n* = 22 (%)	**Fibrosis F3-F4** *n* = 27 (%)		
TT	5 (22.7)	10 (37)	0.840 (0.225-3.137)	0.532
CC+CT	17 (77.3)	17 (63)		
